# Evaluation of three force-position hybrid control methods for a robot-based biological joint-testing system

**DOI:** 10.1186/s12938-016-0195-9

**Published:** 2016-06-07

**Authors:** Hong-Jung Hsieh, Chih-Chung Hu, Tung-Wu Lu, Hsuan-Lun Lu, Mei-Ying Kuo, Chien-Chung Kuo, Horng-Chaung Hsu

**Affiliations:** Institute of Biomedical Engineering, National Taiwan University, No. 1, Sec. 1, Jen-Ai Road, Taipei, 100 Taiwan, R.O.C; Department of Mechanical and Automation Engineering, Kao Yuan University, Kaohsiung, Taiwan; Department of Mechanical Engineering, Ming Chi University of Technology, Taipei, Taiwan; Department of Orthopaedic Surgery, School of Medicine, National Taiwan University, Taipei, Taiwan; Department of Physical Therapy, China Medical University, Taichung, Taiwan; Department of Orthopaedics, China Medical University Hospital, Taichung, Taiwan

## Abstract

**Background:**

Robot-based joint-testing systems (RJTS) can be used to perform unconstrained laxity tests, measuring the stiffness of a degree of freedom (DOF) of the joint at a fixed flexion angle while allowing the other DOFs unconstrained movement. Previous studies using the force-position hybrid (FPH) control method proposed by Fujie et al. (J Biomech Eng 115(3):211–7, 1993) focused on anterior/posterior tests. Its convergence and applicability on other clinically relevant DOFs such as valgus/varus have not been demonstrated. The current s1tudy aimed to develop a 6-DOF RJTS using an industrial robot, to propose two new force-position hybrid control methods, and to evaluate the performance of the methods and FPH in controlling the RJTS for anterior/posterior and valgus/varus laxity tests of the knee joint.

**Methods:**

An RJTS was developed using an industrial 6-DOF robot with a 6-component load-cell attached at the effector. The performances of FPH and two new control methods, namely force-position alternate control (FPA) and force-position hybrid control with force-moment control (FPHFM), for unconstrained anterior/posterior and valgus/varus laxity tests were evaluated and compared with traditional constrained tests (CT) in terms of the number of control iterations, total time and the constraining forces and moments.

**Results:**

As opposed to CT, the other three control methods successfully reduced the constraining forces and moments for both anterior/posterior and valgus/varus tests, FPHFM being the best followed in order by FPA and FPH. FPHFM had root-mean-squared constraining forces and moments of less than 2.2 N and 0.09 Nm, respectively at 0° flexion, and 2.3 N and 0.14 Nm at 30° flexion. The corresponding values for FPH were 8.5 N and 0.33 Nm, and 11.5 N and 0.45 Nm, respectively. Given the same control parameters including the compliance matrix, FPHFM and FPA reduced the constraining loads of FPH at the expense of additional control iterations, and thus increased total time, FPA taking about 10 % longer than FPHFM.

**Conclusions:**

The FPHFM would be the best choice among the methods considered when longer total time is acceptable in the intended clinical applications. The current results will be useful for selecting a force-position hybrid control method for unconstrained laxity tests using an RJTS.

## Background

Measurement of the stiffness of a human joint is clinically relevant and has been a focus of biomechanical research over the last few decades [1–6]. The stiffness of a joint is a biomechanical measure to quantify the clinically termed joint laxity or joint stability. Stability or laxity of a joint depends on the structural properties of the passive tissues, mainly the ligaments, joint capsules and articular surfaces. Measurement of joint stiffness thus helps to detect the condition of the passive tissues, especially the ligaments [7–9].

Previous studies have used testing rigs with a single degree of freedom (DOF) [10–12] or multiple DOFs [13–15] for measuring joint laxity and/or stability. With the femur fixed, the tibia was displaced along the anterior-posterior (AP) axis at a fixed flexion angle, and the applied forces and the associated displacements were used to obtain the non-linear AP stiffness of the joint. For such AP laxity tests, constraining forces and moments were required for flexion/extension (FE), valgus/varus (VV) and internal/external (IE) rotations, and proximal/distal (PD) and lateral/medial (LM) translations. This type of test is here referred to as a constrained test (CT) (e.g., [15]). While the control of CT is relatively straightforward, it does not replicate the manual drawer test in which the tibia is free to move while being displaced along the AP (primary) direction at a fixed flexion angle without applying additional constraining forces and moments to stabilize the other secondary DOFs (i.e., PD and LM translations, and VV and IE rotations). The constraining forces and moments during CT are also expected to affect the measured joint stiffness. To mimic manual laxity tests using 6-DOF test systems, control methods are needed to apply testing loads and displacements without constraining forces and moments in the other secondary DOFs [16, 17]. These types of tests are here referred to as unconstrained tests and the associated control methods as unconstrained control methods. It is also important that a testing system be capable of reproducing the intact knee motion after transection of a ligament of interest so that the in situ force carried by the ligament can be determined as the difference in the joint resultant forces before and after resection [10, 18].

**Fig. 1:**
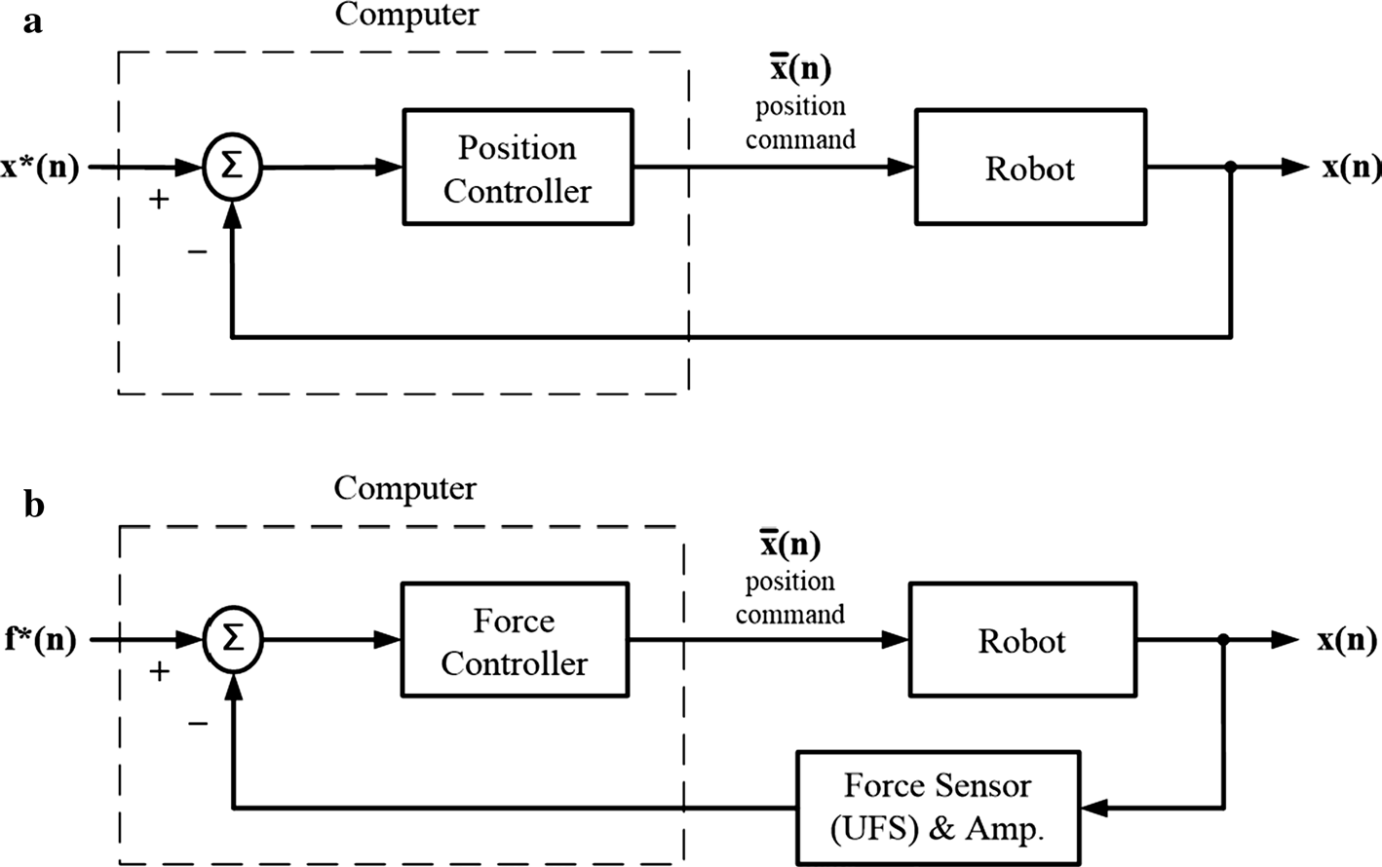
Block diagrams for industrial robot control under position commands. Position and force control methods for industrial robots controlled under position commands: **a** Position control: for a targeted position, the position command x* is input to the position controller which takes the position difference between x* and the real position *x* fed back from the robot to give the new position command $\bar{x}$. **b** Force control: for a targeted force (moment), the force controller takes the force difference between f* and the real force (moment) *f* fed back from the force sensor attached to the robot to give the new position command $\bar{x}$

Among common closed-loop control methods, position control is a basic technique for positioning/tracking tasks [19, 20]. In position control, for industrial robots controlled by position commands, the position of the robot is fed back to the position controller, which generates the new position command to the robot (Fig. 1a). For a targeted position, the position command x* is input to the position controller which takes the position difference between x* and the real position *x* fed back from the robot to give the new position command $\bar{x}$. Laxity tests using pure position control cannot be guaranteed to be free from the constraining forces and moments. Another approach is via force feedback control, in which the forces and moments of the effector are measured and fed back to the force controller, which subsequently generates the new position command to the robot (Fig. 1b). For a targeted force (moment), the force controller takes the force difference between f* and the real force (moment) *f* fed back from the force sensor attached to the robot to give the new position command $\bar{x}$. With pure force control, the correct tracking of the tibia may not be guaranteed.

**Fig. 2:**
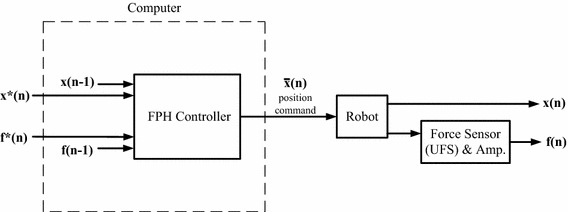
The control block diagram of the force-position hybrid control (FPH). Force-position hybrid control (FPH) for industrial robots with 6-component force sensors (UFS) attached at the effector, and controlled under position commands as proposed by Fujie et al. [17]. It is a stepwise method that for the targeted position x* and force f* at the *nth* increment (motion step) the controller takes the position *x(n*−*1)* (fed back from the robot) and the residual constraining forces and moments *f(n*−*1)* (fed back from the UFS) of the previous increment to determine the displacement for the next increment $\bar{x}(n)$ using a prescribed compliance matrix (C-matrix) [17], aimed at bringing the constraining forces and moments to zero (f*(n)) at the next step. Note that no position or force feedback was used within a step, i.e., no control iterations within the increment

Given the limitations of pure position and pure force control for multi-DOF test apparatus, Fujie et al. [17] first proposed a 6-DOF industrial robot with a force-position hybrid (FPH) control method for an unconstrained AP laxity test of the knee. This approach has also been used for assessing the stiffness and/or ligament forces in other joints [4, 21]. Force-position hybrid control methods aim to reduce the non-zero constraining forces involved in the pure position control method, and the position tracking errors involved in the pure force control method simultaneously, enabling a 6-DOF robot-based system to simulate a clinical unconstrained laxity test. The FPH control method performs an unconstrained AP laxity test by forcing an AP displacement at a fixed flexion angle (i.e., position control in the AP and FE directions) while allowing the tibia the freedom to move in the other DOFs to reduce constraining forces and moments (i.e., force-moment control in the other secondary DOFs). It is a stepwise method that takes the position *x*(*n*−*1*) and residual constraining forces and moments *f*(*n*−*1*) of the previous motion step and determines the displacements for the next motion step $\bar{x}(n)$ to reduce the residual constraining forces and moments (Fig. 2). No position or force feedback is used within a step. More specifically, during a given motion step of the FPH, the non-zero constraining forces and moments in the four secondary DOFs are used to determine the poses of the joint at the next step using an estimated compliance matrix (C-matrix), aimed at bringing the constraining forces and moments to zero at the next step. Any non-zero constraining forces and moments at the next step are then used to determine the following step, and so on. However, owing to the inaccuracy of the compliance matrix, there are inevitably non-zero constraining forces and moments at all steps of the test trajectory. This could potentially affect the accuracy of the test and the measured joint stiffness.

Another limitation of the force-position hybrid control method [17] is that the tests are restricted to stepwise quasi-static loading with a relatively low loading rate (Fujie et al. and related papers listed by Lawless [27]). Furthermore, velocity-based force control methods [22] may also have limitations on loading rates subject to the robot jog buffer configuration. To improve the loading rate, Lawless proposed an adapted stiffness velocity-based force control method and applied it to a hexapod [23]. It is noted that in order to allow a high-rate force-position control, the structure of the manipulator has to be very rigid and the precision has to be very high, which is often difficult to achieve in commercially available articulated manipulators. Therefore, self-designed mechanisms/structures are needed [22, 23]. For example, Fujie et al. [24] developed a robotic system of rigid body/structure that allows high-rate force-position control of the knee joint using a velocity-impedance control. The velocity impedance strategy for the continuous servo system used force control with modified velocity-impedance control, and position control with velocity control [25]. Self-designed systems with high rigidity and precision enable high-rate force-position control for the simulation of more physiological conditions. Therefore, developments towards more advanced high-rate, precise force-position controls continue to be an important area of study. Nonetheless, industrial robots with force-position hybrid control methods at relatively low loading rate are considered sufficient for simulating clinical laxity tests [4, 21, 26], but better strategies are needed for reducing the non-zero constraining forces and moments.

Different combinations of the force and position control strategies in force-position hybrid controls may have different performances in the simulated unconstrained joint laxity tests, i.e., different residual constraining forces and moments. On the other hand, the existing methods use the residual constraining forces and moments at the current step to reduce the constraining forces and moments at the next step, leaving the none-zero constraining forces and moments at the current step unaltered. It is very likely that additional position and/or force-moment control iterations at each step (increment) in the primary DOF may be helpful for reducing the constraining forces and moments for the current position, giving better accuracy and precision. To the best knowledge of the authors, no study has proposed alternative hybrid position-force control methods that incorporate within-step control iterations for unconstrained joint laxity tests using industrial robot-based joint-testing systems and evaluated their performance on testing of the knee joint. Moreover, previous studies have focused mainly on unconstrained AP laxity tests. Its application in evaluating joint stability in other clinically relevant directions such as VV has not been demonstrated in the literature.

The purposes of the current study were to develop a 6-DOF, industrial robot-based joint-testing system, and to propose two new force-position hybrid control methods, namely force-position alternate control (FPA) and Force-Position Hybrid control with force-moment control (FPHFM), and to evaluate the new methods and the Fujie method (FPH) in terms of their stability in controlling the test system for the AP and VV laxity test of the human knee joint.

## Methods

### Ethics statement

A fresh-frozen intact human knee joint was used in the current study. It was obtained from a donor who had undergone an above-knee amputation procedure for reasons other than trauma or disease of the joint. The donor gave informed written consent as approved by the Institutional Research Board of China Medical University Hospital (DMR101-IRB1-139 (CR-2)).

### Robot-based joint-testing system (RJTS)

**Fig. 3:**
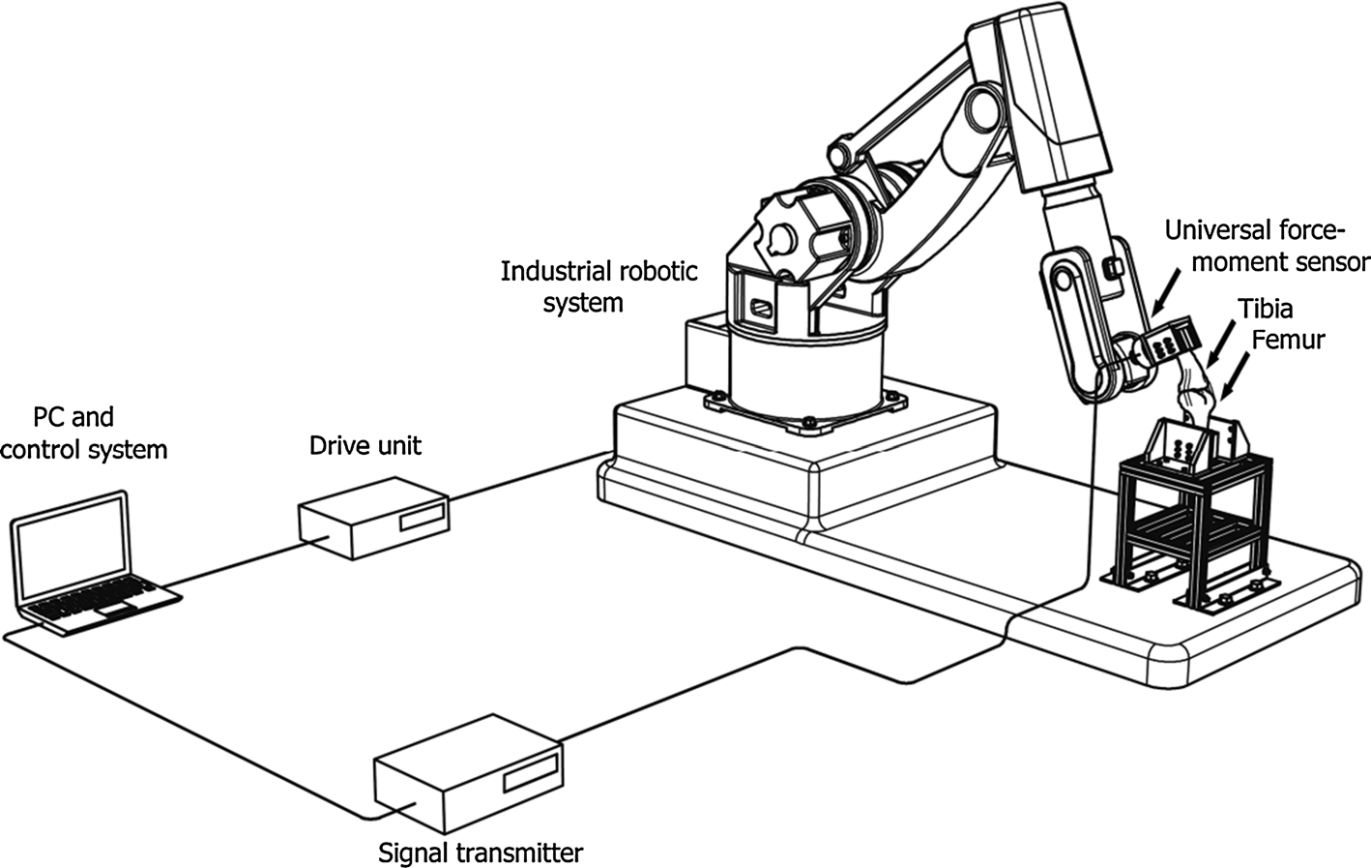
Configuration of the robot-based joint-testing system (RJTS). With the RJTS developed in the current study for testing the knee joint, the tibia was fixed to the 6-component force sensor (UFS) attached to the effector of the robot, while the femur was rigidly fixed to the ground via a metal frame

A robot-based joint-testing system (RJTS) was built for evaluating the control methods (Fig. 3). The RJTS comprised an industrial robotic system (RV-20A, Mitsubishi Electric Corporation, Japan) which provided accurate position control (precision: ± 0.1 mm); a universal force-moment sensor (UFS, Model PY6-100, Bertec Corporation, USA) which was attached to the robot effector to measure three force and three moment components applied to the effector; metallic fixation devices that attached the experimental bone segments to the UFS; as well as a house-developed computer program in Visual Basic for controlling the testing system. The accuracy of the UFS for force and moment components was 1 N and 0.1 Nm, respectively, which was 0.2 % of the nominal maximum loads (500 N for force and 50 Nm for moment).

### Specimen preparation

The RJTS and the associated control methods were evaluated in vitro using the fresh-frozen intact human knee joint. The knee specimen was stored at −70 °C immediately after harvest and was thawed at room temperature 24 h prior to experiment. The knee specimen had a length of 20 cm proximal and 20 cm distal to the joint line. Prior to test, the skin and muscles of the specimen were removed, and the tibia and femur were secured with screws to the UFS and a metallic base fixed to the ground, respectively.

### Coordinate system

**Fig. 4:**
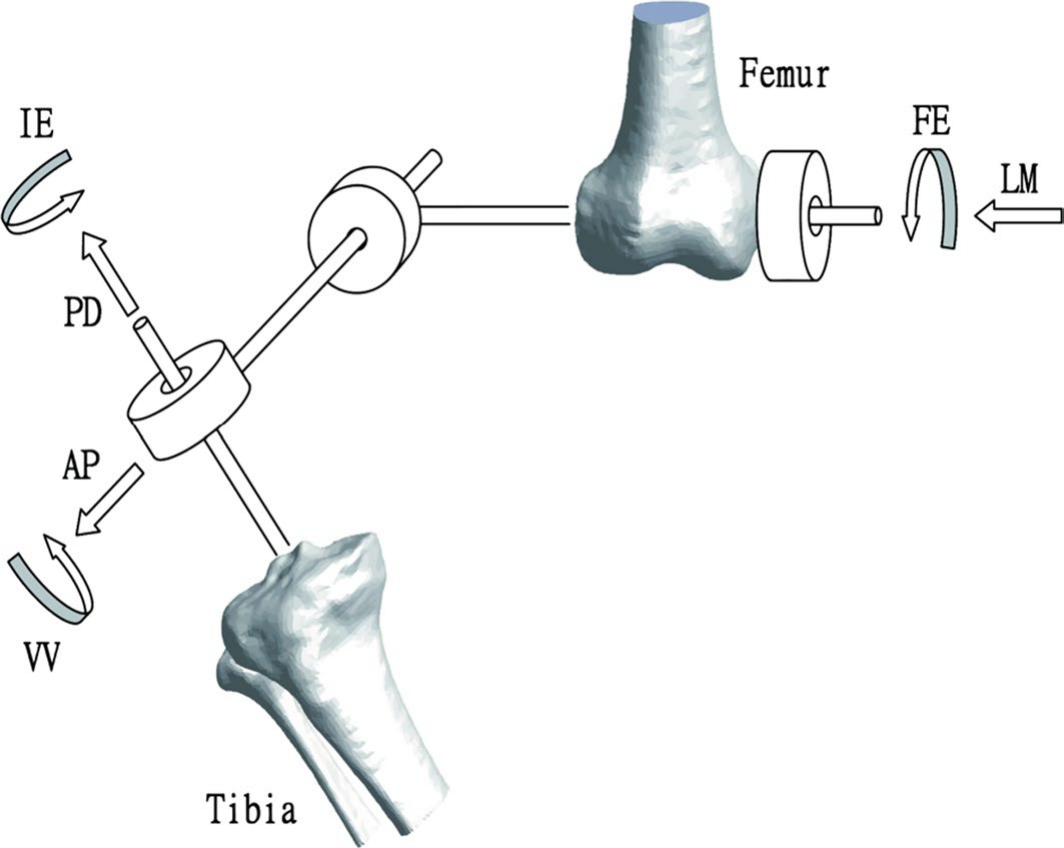
Definition of the joint coordinate system (JCS) of the knee. The definition of the JCS was based on the bone-embedded coordinate systems of the tibia and femur, adapted from Grood and Suntay [27]. The terms for each component of the knee motion are also given

Once the specimen was positioned in the required test pose, coordinates of landmarks on the bones gathered during a calibration trial were used to define the anatomical coordinate system of each of the bones, with the positive x-axis directed anteriorly, the positive y-axis superiorly and the positive z-axis to the right. The knee joint coordinate system (JCS) was then defined based on the anatomical coordinate systems of the bones following a z–x–y Cardanic rotation sequence [27], relative to which the AP, PD and LM translations, as well as the VV, IE and FE rotations, were described (Fig. 4).

### General procedure of force-position hybrid control

**Fig. 5:**
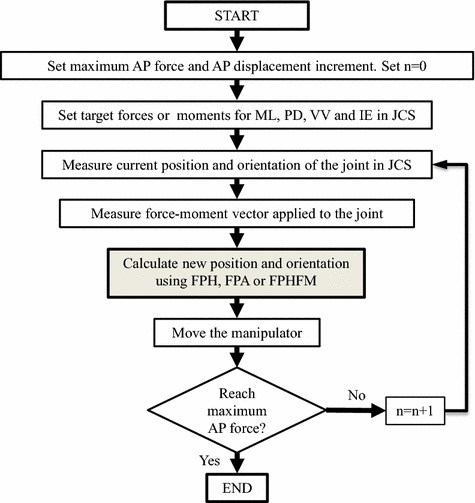
General flow of force-position hybrid control for laxity tests. At the beginning of the test, the maximum AP force and AP displacement increment, as well as the target forces or moments for the secondary DOFs (i.e., ML, PD, VV and IE), are set first. The RJTS then measures the current position and orientation of the knee joint, as well as the force-moment vector applied to the joint, from which the new position and orientation of the knee are calculated and new position commands to the robot are generated using FPH, FPA or FPHFM. If the AP force does not reach the maximum AP force, the control continues to the next step. Laxity tests on other DOFs follow the same general procedure

Based on the RJTS, the general flow of the force-position hybrid control with the AP laxity test as an example is described in Fig. 5. At the beginning of the test, the maximum AP force and AP displacement increment, as well as the target forces or moments for the secondary DOFs (i.e., ML, PD, VV and IE), are set first. The RJTS then measures the current position and orientation of the knee joint, as well as the force-moment vector applied to the joint, from which the new position and orientation of the knee are calculated and new position commands to the robot are generated using FPH, FPA or FPHFM. If the AP force does not reach the maximum AP force, the control continues to the next step. Laxity tests on other DOFs follow the same general procedure.

For the AP laxity test (drawer test) of the knee, the robot was controlled to move the tibia anteriorly through a sequence of infinitesimal displacements (incremental displacements or motion steps of the robot) at the required flexion angle without constraining forces and moments in the other secondary DOFs. The infinitesimal anterior tibial displacement from the current pose was described using a differential translation and rotation transformation (DTRT) [17, 28] as follows.

1 $${\text{DTRT = }}\left[ {\begin{array}{*{20}c} 1 & 0 & 0 & {\partial d_{x} } \\ 0 & 1 & 0 & {\partial d_{y} } \\ 0 & 0 & 1 & {\partial d_{z} } \\ 0 & 0 & 0 & 1 \\ \end{array} } \right] \, \cdot \left[ {\begin{array}{*{20}c} 1 & { - \partial \theta_{z} } & {\partial \theta_{y} } & 0 \\ {\partial \theta_{z} } & 1 & { - \partial \theta_{x} } & 0 \\ { - \partial \theta_{y} } & {\partial \theta_{x} } & 1 & 0 \\ 0 & 0 & 0 & 1 \\ \end{array} } \right] - I\, = \,\left[ {\begin{array}{*{20}c} 0 & { - \partial \theta_{z} } & {\partial \theta_{y} } & {\partial d_{x} } \\ {\partial \theta_{z} } & 0 & { - \partial \theta_{x} } & {\partial d_{y} } \\ { - \partial \theta_{y} } & {\partial \theta_{x} } & 0 & {\partial d_{z} } \\ 0 & 0 & 0 & 0 \\ \end{array} } \right]$$

where *δd*_*x*_, *δd*_*y*_ and *δd*_*z*_ are differential translations along, and *δθ*_*x*_, *δθ*_*y*_ and *δθ*_*z*_ are differential rotations about the x-, y-, and z-axes of the tibial coordinate system, respectively [28]. To keep the constraining forces and/or moments at the secondary DOFs close to zero as much as possible, the infinitesimal anterior tibial displacement for the next motion step (increment) was determined considering a force-moment control procedure. For this purpose, the force and moment vector *F* at the knee in the JCS was calculated from the force and moment vector ^*s*^*F* measured by the UFS in the sensor coordinate system as follows [27, 29].

2 $$F = (J_{tk}^{ - 1} J_{st}^{ - 1} )^{s} F$$

where *J*_*tk*_ was the Jacobian from the tibial coordinate system to the JCS of the knee, and *J*_*st*_ was the Jacobian from the sensor coordinate system to the JCS of the knee. Detailed derivations of these Jacobian matrices can be found in Paul [28] and Fujie et al. [29]. Given the difference of the constraining force-moment vector *F*_*d*_ between the measured force-moment vector and the target—zero in the current case—the differential motion *D* in the JCS could be obtained using a six-by-six compliance matrix *C* as follows.

3 $$D = CF_{d}$$

The compliance matrix C used in the current study followed that proposed by Fujie et al. [30] for fast force-moment control while avoiding mechanical vibration of the system.

4 $${\text{C}} = \left[ {\begin{array}{*{20}c} 0 & 0 & 0 & 0 & 0 & 0 \\ 0 & {1.0} & 0 & 0 & 0 & 0 \\ 0 & 0 & {0.15} & 0 & 0 & 0 \\ 0 & 0 & 0 & {1.0} & 0 & 0 \\ 0 & 0 & 0 & 0 & {0.15} & 0 \\ 0 & 0 & 0 & 0 & 0 & {1.0} \\ \end{array} } \right]$$

### Three hybrid control methods

**Fig. 6:**
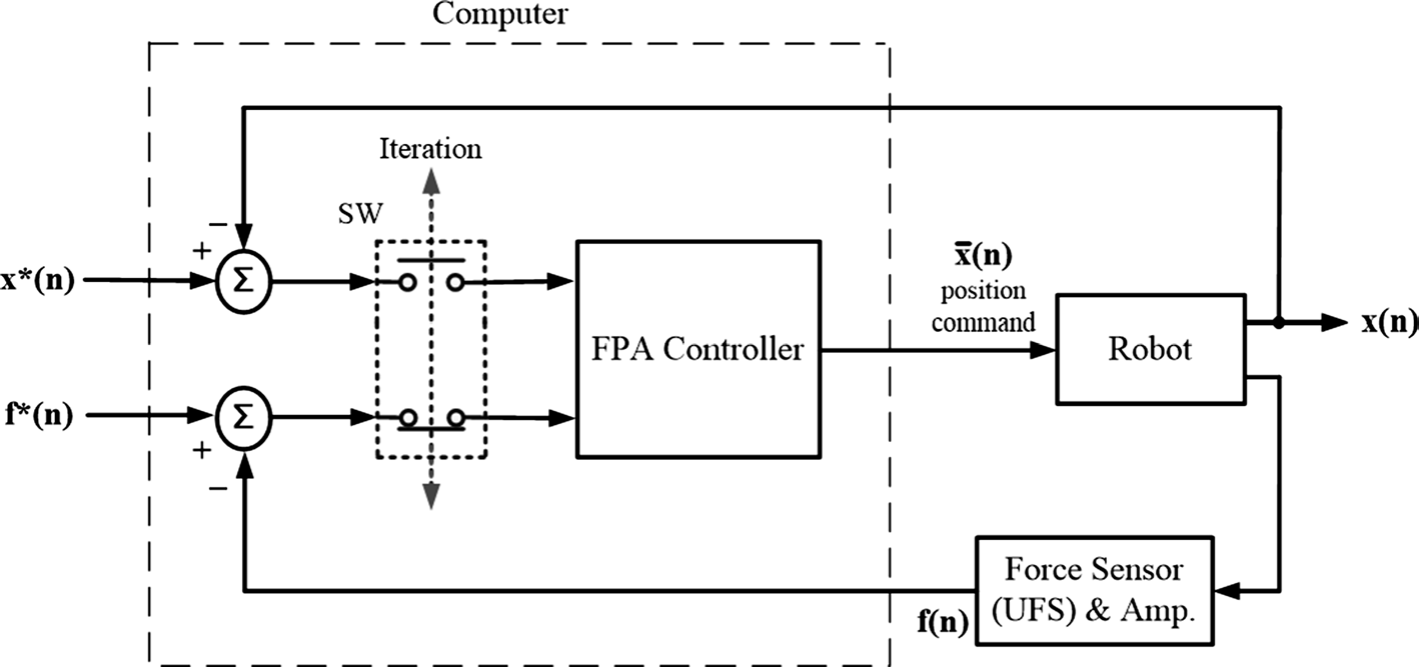
The control block diagram of the force-position alternate control (FPA). Force-position alternate control (FPA) proposed for industrial robots with 6-component force sensors (UFS) attached at the effector and controlled under position commands. For the targeted position x*(n) and force f*(n) at the *nth* increment, the FPA controller takes alternately the position difference between x*(n) and the real position *x(n)* (fed back from the robot), and the force difference between f*(n) and the real force *f(n)* (fed back from the UFS) to give the new position command $\bar{x}(n)$. The feedback iterations continue until the position and force errors are within an acceptable tolerance, at which point the test will continue to the next increment as described in the general procedure. The alternate input into the controller for each iteration is controlled by switch SW

**Fig. 7:**
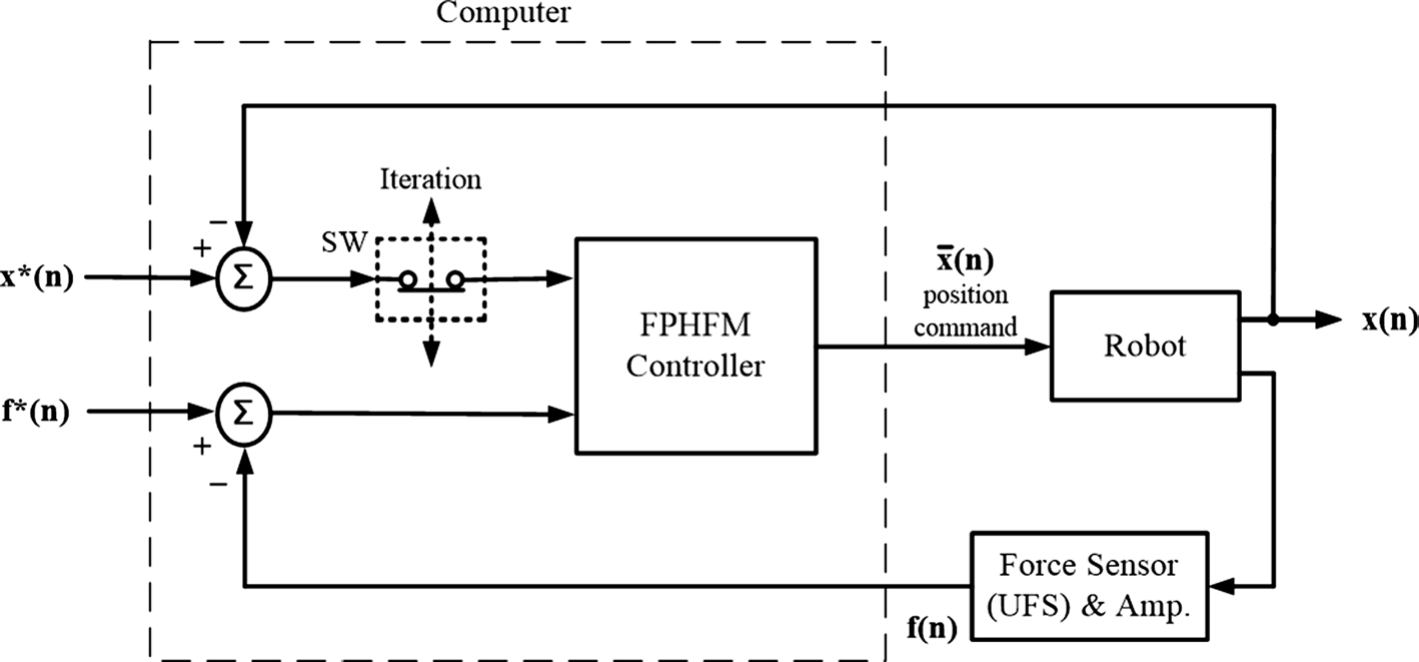
The control block diagram of the force-position hybrid control with force-moment control (FPHFM). Force-position hybrid control with force-moment control (FPHFM) proposed for industrial robots with 6-component force sensors (UFS) attached at the effector and controlled under position commands. For the targeted position x*(n) and force f*(n) at the *nth* increment, the FPHFM controller switches between FPH and force control. In other words, for one control iteration the position difference between x*(n) and the real position *x(n)* (fed back from the robot) and the force difference between f*(n) and the real force *f(n)* (fed back from the UFS) are used as in FPH, and for the next iteration the force difference between f*(n) and the real force f(n) will be used as in pure force control. The input for the FPH and the input for the force control is controlled by the SW

Based on the above-described general procedure, the FPH method [17] and two new methods, namely force-position alternate control (FPA) and force-position hybrid control with force-moment control (FPHFM), for unconstrained laxity tests were implemented on the RJTS. The three methods differed primarily in how the position and force/moment errors were fed back to the controller to determine the position and orientation of the knee for the next motion step (displacement increment). The FPH method did not have a feedback loop within a displacement increment (Fig. 2) whereas the position and force-moment errors were fed back to the FPA controller alternately within a displacement increment (Fig. 6). In FPHFM, the FPH and a force-moment feedback control were applied alternately within a displacement increment (Fig. 7). The control using each of the two new methods within a displacement increment is described briefly as follows.

Within a displacement increment (motion step) under FPA control, for the targeted position x*(n) and force f*(n), the hybrid force-position alternate controller takes alternately the position difference between x*(n) and the real position *x(n*) fed back from the robot and the force difference between f*(n) and the real force *f(n)* fed back from the UFS to give the new position command $\bar{x}(n)$ (Fig. 6). The alternate input into the controller is controlled by switch SW. Once the position and force errors are within an acceptable tolerance, the test will continue to the next displacement increment as described in the general procedure (Fig. 5).

For the targeted position x*(n) and force f*(n) within a displacement increment, the FPHFM controller switches between FPH and force-moment feedback control (Fig. 7). In other words, for one iteration the position difference between x*(n) and the real position *x(n)* fed back from the robot and the force difference between f*(n) and the real force *f(n)* fed back from the UFS were used (equivalent to FPH), and for the next iteration the force difference between f*(n) and the real force *f(n)* fed back from the UFS would be used (equivalent to force control). The input for the FPH and the input for the Force Control were used alternately and controlled by the SW (Fig. 7).

The three control methods were used to perform unconstrained AP and VV laxity tests of the knee specimens at 0**°** and 30**°** flexion. For comparison, the specimen was also tested using the constrained testing (CT) method, under which the tibia was displaced precisely along a prescribed axis under position control while the constraining forces and moments in the secondary DOFs were measured (i.e., PD and LM forces, and VV and IE moments during AP tests; and AP, PD and LM forces and IE moment during VV tests).

### Unconstrained AP laxity test

For the unconstrained AP laxity test using FPH, the tibia of the cadaveric knee was displaced anteriorly/posteriorly by the RJTS by pre-determined increments (0.2 mm in the current study) to reach maximum AP force under position control in the AP and FE directions, and force-moment control of the PD and LM forces, and VV and IE moments (Fig. 5). At a given incremental position, the non-zero PD and LM forces, and VV and IE moments (i.e., *F*_*d*_) were used, together with position data, to calculate the DTRT for the next increment by a proper choice of the C-matrix (Fig. 2; Eq. 4).

With FPA, the tibia was displaced anteriorly/posteriorly by the same pre-determined increment to reach maximum AP force, and for each increment the pose of the knee was fine-tuned under position and force control alternately to reduce *F*_*d*_ to be within a small tolerance (1.2 N for force components, 0.12 Nm for moment components in the current study) (Figs. 5, 6).

With FPHFM, the tibia was displaced by the pre-determined increment to reach the maximum AP force, and for each increment the FPH and force-moment feedback control was used alternately to reduce *F*_*d*_ until within the same tolerance (Figs. 5, 7).

### Unconstrained VV laxity test

For the unconstrained VV laxity test, the tibia of the cadaveric knee was rotated about the VV axis by the RJTS by pre-determined increments (0.1° in the current study) to reach maximum moments (±4.0 Nm) under position control of VV and FE, and force-moment control of the AP, PD and LM forces, and the IE moment (Fig. 5). With this common basis, the three hybrid control methods, namely FPH, FPA and FPHFM, were used to deal with the non-zero constraining forces and moments following strategies similar to those used for the unconstrained AP laxity tests.

### Experimental procedure

To avoid sequence dependences of the results, the order of testing was randomized for each test condition. For example, for the AP test at 0° of knee flexion, three measurement trials for each control method were performed, resulting in a total of 12 trials. The order of these 12 trials was randomized. Preconditioning and breaks between trials were also used. Following Lee et al. [31], at the beginning of the experiment, a passive path during knee flexion-extension between 0° and 120° was defined, and each of the subsequent trials began with preconditioning the knee by flexing and extending it ten times through the passive path. Between the test trials, there was a 5-minute break before the preconditioning, and the specimen was prevented from dehydrating by putting saline-soaked scarves on the surface of the specimen. The specimen was also sprayed regularly with 0.9 % saline to avoid tissue dehydration during the experiment.

### Data analysis

For the evaluation of the control methods, the means and standard deviations of the number of increments, number of control iterations, and total time for each test condition and each method were obtained. The means and standard deviations of the root-mean-squared (RMS) values of each of the constraining force and moment components were also calculated for the converged incremental steps for each test condition and each method.

## Results

### Number of increments, total number of iterations and total time

**Fig. 8:**
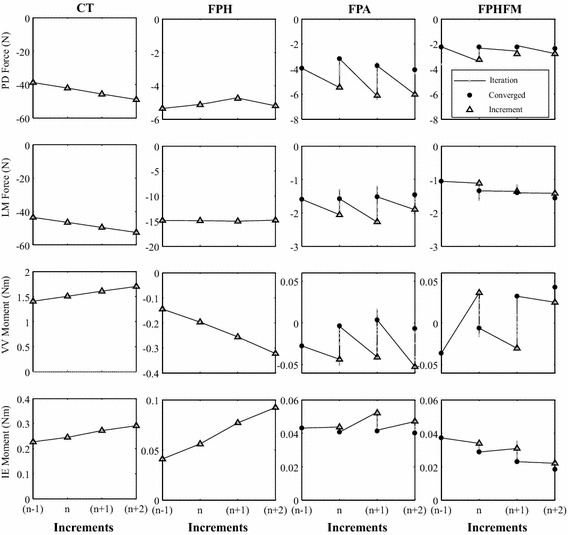
Control iterations at test increments for all control methods during the AP test. Changes of the constraining force (PD and LM) and moment (VV and IE) components with control iterations (*grey lines*) at three typical test increments for each of the control methods during the AP test. No control iterations were used for CT and FPH, so their constraining force and moment values were the incremental values (*black triangles*). For FPA and FPHFM, the incremental values (*black triangles*) were further reduced over a number of control iterations (*grey dots*) and converged to the final values (*black dots*)

**Fig. 9:**
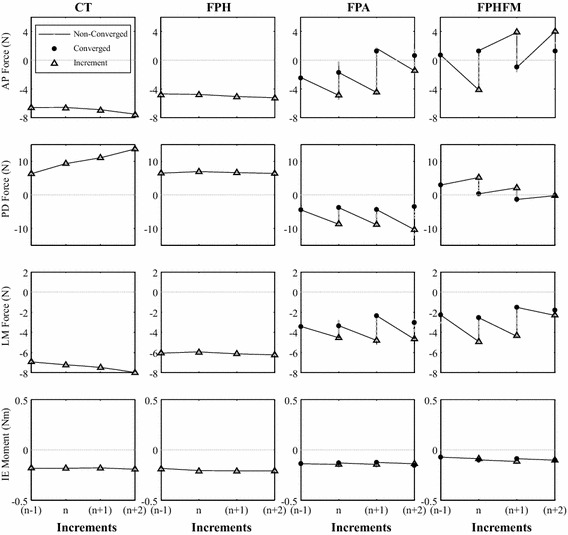
Control iterations at test increments for all control methods during the VV test. Changes of the constraining force (AP, PD and LM) and moment (IE) components with control iterations (*grey lines*) at three typical test increments for each of the control methods during the VV test. No control iterations were used for CT and FPH, so their constraining force and moment values were the incremental values (*black triangles*). For FPA and FPHFM, the incremental values (*black triangles*) were further reduced over a number of control iterations (*grey dots*) and converged to the final values (*black dots*)

Since no feedback iterations were needed for CT and FPH (Figs. 8, 9), CT was found to use the shortest total time (123 ± 1 s) for the smallest number of increments (189 ± 1) for AP tests while FPH was found to use the shortest total time (78 ± 2 s) for the smallest number of increments (98 ± 1) for VV tests (Table 1). On the other hand, to reduce constraining forces and moments both FPA and FPHFM needed a larger number of control iterations (Figs. 8, 9), and thus had a total time for the number of increments similar to CT and FPH (Table 1). FPA used a greater number of control iterations and total time than FPHFM, for both AP and VV tests (Table 1).

**Table 1:** Control increments, iterations and total time during constrained and unconstrained tests

Control method	AP test						VV test					
	0° flexion			30° flexion			0° flexion			30° flexion		
	N	n	T (s)	N	n	T (s)	N	n	T (s)	N	n	T (s)
CT	189 (1)	189 (1)	123 (1)	191 (1)	191 (1)	120 (0)	101 (4)	101 (4)	80 (2)	117 (1)	117 (1)	96 (0)
FPH	211 (1)	211 (1)	141 (1)	205 (4)	205 (4)	129 (2)	98 (5)	98 (5)	78 (2)	115 (0)	115 (0)	95 (0)
FPA	239 (2)	1135 (28)	689 (11)	214 (2)	1001 (8)	590 (3)	113 (1)	1164 (11)	680 (4)	127 (0)	1139 (9)	596 (4)
FPHFM	267 (10)	1024 (30)	615 (11)	210 (8)	844 (24)	520 (10)	112 (2)	1064 (15)	635 (6)	125 (1)	1110 (3)	577 (1)

Means (standard deviations) of the number of increments, total number of control iterations and total time during the constrained test (CT), and unconstrained tests using force-position hybrid control (FPH), force-position alternate control (FPA), and force-position hybrid control & force-moment control (FPHFM) for AP and VV tests at 0° and 30° knee flexion. CT and FPH needed a similar number of control iterations (total time) while FPHFM needed more and FPA needed even more

N, number of increments; n, total number of control iterations; T, total time

### Residual constraining forces and moments

#### Constrained AP test

**Fig. 10:**
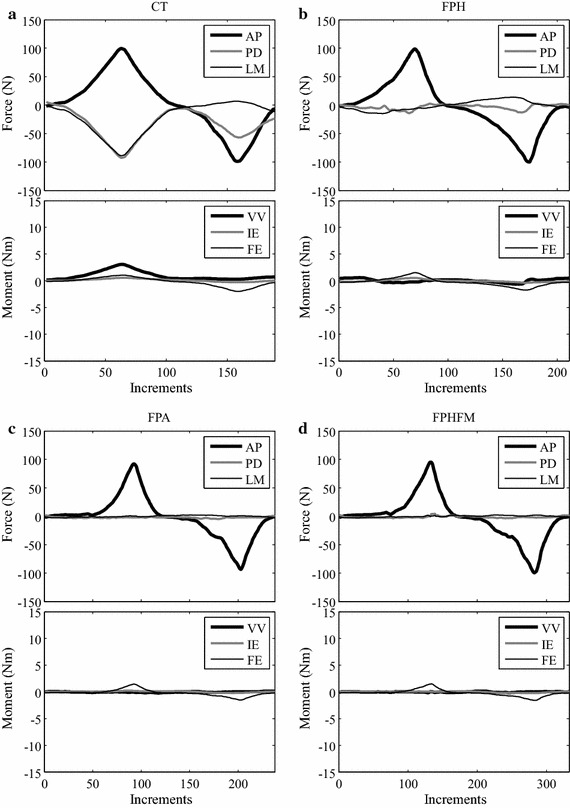
Histories of the force and moment components during the AP test at 0° knee flexion. Changes of all the force and moment components during the constrained AP test using CT (**a**), and unconstrained AP tests using FPH (**b**), FPA (**c**) and FPHFM (**d**). While the constraining forces (PD and LM) and moments (VV and IE) were quite large during CT with RMS values of up to 38.4 N and 0.97 Nm, respectively, their values were greatly reduced during unconstrained tests (**b**–**d**). The FPHFM and FPA were able to keep the constraining forces and moments close to the measurement accuracy, with RMS values of less than 2.4 N and 0.11 Nm, respectively, and the RMS values of FE angles less than 1.36°. The FPH had RMS values of up to 8.5 N and 0.33 Nm for the constraining force and moment components, respectively

**Fig. 11:**
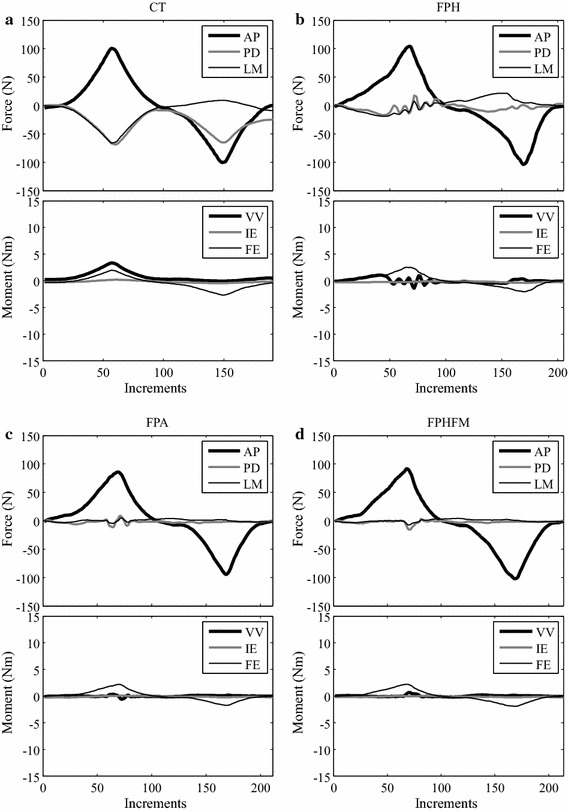
Histories of the force and moment components during the AP test at 30° knee flexion. Changes of all the force and moment components during the constrained AP test (**a** CT), and unconstrained AP tests using (**b**) FPH, (**c**) FPA and (**d**) FPHFM. While the constraining forces (PD and LM) and moments (VV and IE) were quite large during CT with RMS values of up to than 35.4 N and 0.82 Nm, respectively, their values were greatly reduced during unconstrained tests (**b**–**d**). The FPHFM and FPA were able to keep the constraining forces and moments close to the measurement accuracy, with RMS values of less than 2.8 N and 0.15 Nm, respectively, and the RMS values of FE angles less than 1.55°. The FPH had RMS values of up to 11.5 N and 0.45 Nm for the constraining force and moment components, respectively

During the AP tests under AP forces of up to ± 100 N using CT, significant constraining forces and moments were needed to maintain accurate tibial translation along the AP axis (Figs. 10, 11). At 0° flexion, the largest constraining force was the PD component with an RMS value of 38.4 ± 1.4 N, and the largest constraining moment was the VV component with an RMS value of 0.97 ± 0.02 Nm (Table 2). Similarly, at 30° flexion, the biggest constraining force was the PD component with an RMS value of 35.4 ± 1.2 N, and the biggest constraining moment was the VV component with an RMS value of 0.82 ± 0.04 Nm (Table 2). The FE angles were maintained accurately at the prescribed testing angles, with RMS errors of less than 0.11 ± 0.00**°**, requiring an RMS FE moment of 1.13 ± 0.00 Nm. For both 0° and 30° flexion, most of the constraining forces and moments during anterior drawer tests were larger than those during posterior tests (Table 2).

**Table 2:** RMS values of the constraining forces and moments during AP tests

Control method	0° Knee flexion						30° Knee flexion					
	Force (N)		Moment (Nm)			Angle (deg)	Force (N)		Moment (Nm)			Angle (deg)
	PD	LM	VV	IE	FE		PD	LM	VV	IE	FE	
Total												
CT	38.4 (1.4)	29.4 (0.7)	0.97 (0.02)	0.26 (0.03)	0.72 (0.01)	0.11 (0.00)	35.4 (1.2)	17.1 (0.4)	0.82 (0.04)	0.26 (0.02)	1.13 (0.00)	0.11 (0.00)
FPH	4.6 (0.4)	8.5 (0.4)	0.33 (0.00)	0.20 (0.03)	0.75 (0.02)	0.14 (0.03)	7.5 (0.3)	11.5 (0.4)	0.45 (0.00)	0.23 (0.01)	1.00 (0.02)	0.40 (0.03)
FPA	2.4 (0.3)	1.4 (0.2)	0.07 (0.00)	0.11 (0.02)	0.51 (0.04)	0.89 (0.03)	2.8 (0.2)	2.1 (0.5)	0.11 (0.01)	0.15 (0.02)	0.82 (0.03)	1.29 (0.01)
FPHFM	2.2 (0.2)	1.4 (0.3)	0.07 (0.03)	0.09 (0.02)	0.50 (0.05)	1.36 (0.19)	2.3 (0.2)	2.2 (0.3)	0.12 (0.00)	0.14 (0.01)	0.95 (0.02)	1.55 (0.02)
Anterior												
CT	48.0 (3.2)	48.0 (0.6)	1.56 (0.06)	0.31 (0.08)	0.54 (0.02)	0.11 (0.00)	34.1 (1.0)	32.7 (0.1)	1.50 (0.01)	0.16 (0.03)	0.78 (0.00)	0.11 (0.00)
FPH	5.8 (0.7)	9.2 (0.7)	0.34 (0.02)	0.27 (0.05)	0.60 (0.07)	0.11 (0.03)	9.8 (1.2)	11.5 (0.4)	0.65 (0.01)	0.22 (0.02)	1.09 (0.02)	0.27 (0.03)
FPA	2.4 (0.2)	1.5 (0.2)	0.07 (0.03)	0.09 (0.03)	0.36 (0.12)	0.46 (0.03)	3.6 (0.2)	2.1 (0.4)	0.12 (0.01)	0.15 (0.01)	0.91 (0.03)	1.20 (0.01)
FPHFM	2.4 (0.2)	1.6 (0.5)	0.07 (0.03)	0.05 (0.02)	0.36 (0.04)	0.65 (0.19)	3.2 (0.1)	2.3 (0.4)	0.13 (0.01)	0.11 (0.01)	1.04 (0.03)	1.44 (0.02)
Posterior												
CT	35.2 (0.7)	4.4 (1.4)	0.41 (0.03)	0.17 (0.01)	1.14 (0.01)	0.11 (0.00)	35.7 (1.5)	5.4 (1.5)	0.26 (0.01)	0.33 (0.02)	1.41 (0.00)	0.11 (0.00)
FPH	3.5 (0.1)	7.9 (0.2)	0.31 (0.01)	0.19 (0.02)	0.83 (0.01)	0.17 (0.03)	5.6 (0.3)	11.5 (0.1)	0.27 (0.00)	0.24 (0.01)	0.99 (0.02)	0.46 (0.03)
FPA	2.4 (0.4)	1.3 (0.1)	0.07 (0.01)	0.13 (0.03)	0.65 (0.02)	1.18 (0.03)	2.3 (0.2)	2.2 (0.7)	0.10 (0.01)	0.15 (0.02)	0.71 (0.04)	1.33 (0.01)
FPHFM	1.9 (0.2)	1.2 (0.2)	0.07 (0.03)	0.14 (0.03)	0.64 (0.05)	1.82 (0.19)	2.2 (0.3)	2.1 (0.3)	0.11 (0.01)	0.15 (0.01)	0.93 (0.02)	1.57 (0.02)

Means (standard deviations) of the RMS values of the constraining forces (PD, ML) and moments (VV, IE) during AP tests of the knee at 0° and 30° flexion during the Constrained Test (CT), and unconstrained tests using Force-Position Hybrid control (FPH), Force-Position Alternate control (FPA), and Force-Position Hybrid control & Force-Moment control (FPHFM). As expected, CT needed the largest constraining forces and moments. For unconstrained tests, FPHFM and FPA achieved much smaller constraining forces and moments than FPH. For both 0° and 30° flexion, most of the constraining forces and moments during anterior tests were larger than those during posterior tests for all methods. The FE moments required to maintain the knee flexion angle are also provided

#### Unconstrained AP test

During unconstrained AP tests at 0**°** and 30**°** knee flexion, the three hybrid control methods successfully varied the AP forces gradually between ± 100 N (Figs. 10, 11). The FPHFM method was able to keep the constraining forces and moments in the secondary DOFs within a small range, with RMS values of less than 2.2 ± 0.2 N and 0.09 ± 0.02 Nm at 0**°** flexion (Fig. 10; Table 2), and less than 2.3 N and 0.14 Nm at 30**°** flexion (Fig. 11; Table 2). The RMS values of FE angles were less than 1.36 ± 0.19**°** and 1.55 ± 0.02**°**, respectively. In contrast, the FPH had RMS values of up to 8.5 ± 0.4 N (about 4 times greater than those of FPHFM) and 0.33 ± 0.00 Nm (about 3 times greater than those of FPHFM) for the constraining force and moment components, respectively, at 0**°** flexion, and 11.5 ± 0.4 N (about 6 times greater than those of FPHFM) and 0.45 ± 0.00 Nm (about 3 times greater than those of FPHFM) at 30**°** flexion (Figs. 10, 11; Table 2). The RMS values of FE angles were less than 0.14 ± 0.03° and 0.40 ± 0.03°, respectively. The performance of FPA was similar to FPHFM, and both were better than FPH (Table 2). For all the three unconstrained control methods, most of the constraining forces and moments during anterior drawer tests were larger than those during posterior tests (Table 2).

#### Constrained VV test

**Fig. 12:**
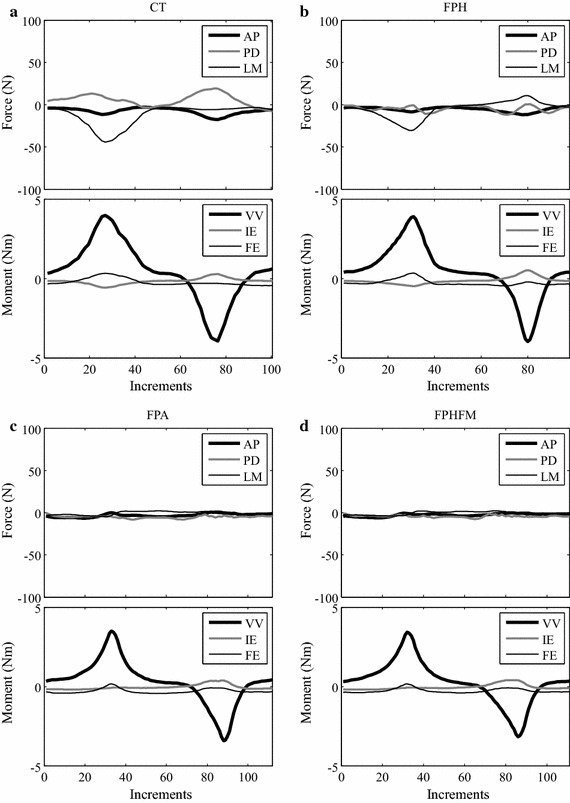
Histories of the force and moment components during the VV test at 0° knee flexion. Changes of all the force and moment components during the constrained VV test using CT (**a**), and unconstrained AP tests using FPH (**b**), FPA (**c**) and FPHFM (**d**). While the constraining forces (AP, PD and LM) and moments (IE) were quite large during CT with RMS values of up to 12.1 N and 0.22 Nm, respectively, their values were greatly reduced during unconstrained tests (**b**–**d**). The FPHFM was able to keep the constraining forces and moments close to the measurement accuracy, with RMS values of less than 4.7 N and 0.13 Nm, respectively, and the RMS values of FE angles less than 0.66°. The FPH had RMS values of up to 8.6 N and 0.23 Nm for the constraining force and moment components, respectively. The values for FPA were in between the FPHFM and FPH

**Fig. 13:**
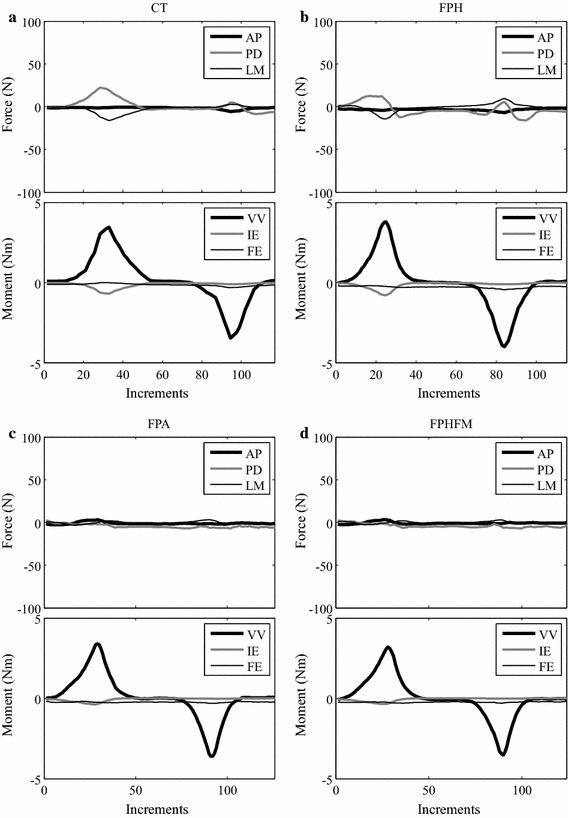
Histories of the force and moment components during the VV test at 30° knee flexion. Changes of all the force and moment components during the constrained VV test (**a** CT), and unconstrained AP tests using (**b**) FPH, (**c**) FPA and (**d**) FPHFM. While the constraining forces (AP, PD and LM) and moments (IE) were quite large during CT with RMS values of up to 9.3 N and 0.22 Nm, respectively, their values were greatly reduced during unconstrained tests (**b**–**d**). The FPHFM was able to keep the constraining forces and moments close to the measurement accuracy, with RMS values of less than 4.9 N and 0.07 Nm, respectively, and the RMS values of FE angles less than 0.28°. The FPH had RMS values of up to 7.9 N and 0.13 Nm for the constraining force and moment components, respectively. The values for FPA were in between the FPHFM and FPH

During the VV tests at 0**°** and 30**°** knee flexion using CT, the VV moments varied gradually between ±4.0 Nm along the defined VV axis (Figs. 12, 13). At 0° flexion, constraining forces and moments were needed to maintain accurate rotation along the VV axis. The largest constraining force was the LM component with an RMS value of 12.1 ± 1.1 N, while the largest constraining moment was the IE component with an RMS value of 0.22 ± 0.00 Nm (Table 3). At 30° flexion, the biggest constraining force was the PD component with an RMS value of 9.3 ± 3.1 N, while an RMS value of 0.22 ± 0.01 Nm was found for the constraining IE moment (Table 3). The FE angles were maintained accurately at the prescribed testing flexion angles with RMS errors less than 0.08 ± 0.01° and 0.13 ± 0.00° under FE moments of 0.31 ± 0.03 Nm and 0.23 ± 0.01 Nm, respectively. Comparable constraining forces and moments were found for varus and valgus tests (Table 3).

**Table 3:** RMS values of the constraining forces and moments during VV tests

Control method		0° Knee flexion						30° Knee flexion					
		Force (N)			Moment (Nm)		Angle (deg)	Force (N)			Moment (Nm)		Angle (deg)
		AP	PD	LM	IE	FE	FE	AP	PD	LM	IE	FE	FE
Total													
CT		7.6 (0.3)	9.0 (2.3)	12.1 (1.1)	0.22 (0.00)	0.31 (0.03)	0.08 (0.01)	2.4 (0.2)	9.3 (3.1)	5.9 (1.2)	0.22 (0.01)	0.23 (0.01)	0.13 (0.00)
FPH		5.7 (0.5)	5.6 (0.4)	8.6 (0.2)	0.23 (0.01)	0.33 (0.01)	0.09 (0.06)	3.4 (0.1)	7.9 (0.5)	2.5 (0.1)	0.13 (0.01)	0.28 (0.01)	0.05 (0.01)
FPA		3.9 (0.1)	5.6 (0.4)	2.3 (0.2)	0.12 (0.00)	0.33 (0.01)	0.54 (0.00)	1.6 (0.1)	5.2 (0.1)	1.2 (0.1)	0.08 (0.01)	0.23 (0.01)	0.36 (0.02)
FPHFM		3.1 (0.4)	4.7 (0.1)	2.2 (0.2)	0.13 (0.00)	0.30 (0.01)	0.66 (0.07)	1.3 (0.1)	4.9 (0.2)	1.1 (0.1)	0.07 (0.01)	0.23 (0.00)	0.28 (0.05)
Valgus													
CT		5.3 (0.5)	7.0 (2.3)	20.2 (0.9)	0.25 (0.02)	0.27 (0.04)	0.05 (0.01)	1.9 (0.3)	11.4 (3.9)	7.4 (1.6)	0.33 (0.01)	0.18 (0.01)	0.06 (0.00)
FPH		4.5 (0.5)	4.4 (1.1)	11.9 (0.4)	0.24 (0.03)	0.31 (0.03)	0.06 (0.06)	3.3 (0.1)	8.5 (0.5)	3.6 (0.1)	0.22 (0.01)	0.25 (0.00)	0.04 (0.01)
FPA		4.7 (0.0)	5.8 (0.6)	2.5 (0.2)	0.12 (0.00)	0.37 (0.01)	0.30 (0.00)	1.8 (0.1)	4.2 (0.1)	1.2 (0.1)	0.13 (0.00)	0.23 (0.01)	0.45 (0.02)
FPHFM		4.0 (0.1)	4.9 (0.2)	2.5 (0.2)	0.13 (0.01)	0.33 (0.02)	0.31 (0.07)	1.9 (0.1)	3.6 (0.2)	1.4 (0.1)	0.19 (0.00)	0.26 (0.00)	0.41 (0.05)
Varus													
CT		11.7 (0.2)	12.1 (1.8)	4.9 (0.7)	0.13 (0.00)	0.36 (0.01)	0.11 (0.01)	4.1 (0.3)	7.9 (3.0)	1.7 (0.9)	0.10 (0.01)	0.34 (0.01)	0.20 (0.00)
FPH		8.2 (0.6)	7.6 (0.3)	4.7 (1.2)	0.21 (0.01)	0.37 (0.01)	0.14 (0.06)	3.6 (0.1)	7.4 (0.5)	2.0 (0.1)	0.06 (0.00)	0.31 (0.00)	0.07 (0.01)
FPA		1.8 (0.1)	5.1 (0.2)	1.7 (0.3)	0.22 (0.00)	0.27 (0.01)	0.78 (0.00)	1.5 (0.1)	6.1 (0.2)	1.3 (0.2)	0.01 (0.01)	0.24 (0.01)	0.23 (0.02)
FPHFM		1.7 (0.4)	4.4 (0.1)	1.7 (0.3)	0.23 (0.01)	0.27 (0.01)	0.94 (0.07)	1.0 (0.1)	5.4 (0.2)	0.9 (0.2)	0.01 (0.01)	0.22 (0.01)	0.22 (0.05)

Means (standard deviations) of the RMS values of the residual constraining forces (AP, PD, ML) and moments (IE) during VV tests of the knee at 0° and 30° flexion during the constrained test (CT), and unconstrained tests using force-position hybrid control (FPH), force-position alternate control (FPA), and force-position hybrid control & force-moment control (FPHFM). As expected, CT needed the largest constraining forces and moments. For unconstrained tests, FPHFM achieved much smaller constraining forces and moments than FPH. The performance of FPA was similar to FPHFM except for AP and PD constraining forces that were in between those of FPHFM and FPH. The FE moments required to maintain the knee flexion angle are also provided

#### Unconstrained VV test

During the unconstrained VV tests, the three hybrid control methods varied the VV moments gradually between ± 4.0 Nm (Figs. 12, 13). At 0° flexion, the FPHFM method was able to keep the constraining forces and moments in the secondary DOFs within a small range (Figs. 12, 13), with RMS values less than 4.7 ± 0.1 N and 0.13 ± 0.00 Nm, respectively (Table 3). The RMS values of FE angles were also small, namely less than 0.66 ± 0.07° under an FE moment of 0.30 ± 0.01 Nm. For 30**°** tests, the maximum RMS values of the residual forces under the control of FPH, FPA and FPHFM were 7.9 ± 0.5 N, 5.2 ± 0.1 N and 4.9 ± 0.2 N, respectively, while the corresponding moments were 0.13 ± 0.01 Nm, 0.08 ± 0.01 Nm and 0.07 ± 0.01 Nm (Table 3).

### Force–displacement curves

**Fig. 14:**
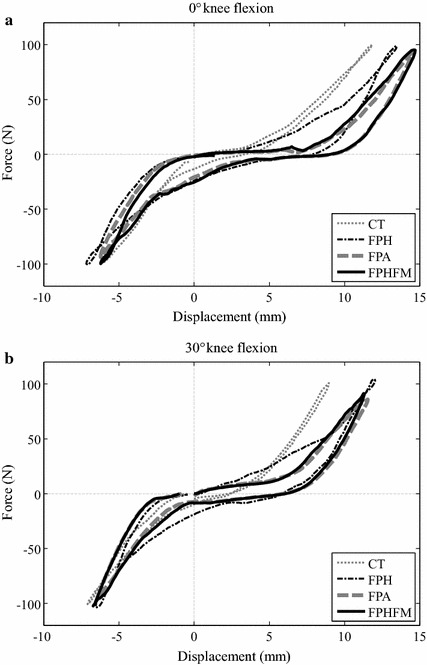
Force-deformation curves during AP tests. The force–deformation curves from the first trial of the constrained AP tests (CT, *thin grey dotted line*) and unconstrained AP tests using FPH (*thin black dash*-*dotted line*), FPA (*thick grey dashed line*) and FPHFM (*thick line*) at (**a**) 0° and (**b**) 30° flexion. In contrast to the posterior stiffness, the anterior stiffness started to differ after 5 mm of anterior deformation while larger anterior stiffness was found using the CT method both at 0° and 30° knee flexion during AP tests. During CT where significant constraining forces and moments were required, the target force was reached with smaller displacement, indicating less laxity, when compared to those of the unconstrained tests using the three unconstrained control methods, especially during the anterior drawer test

**Fig. 15:**
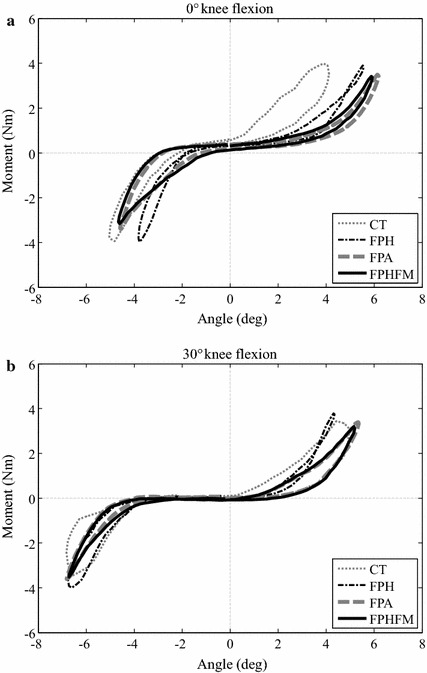
Force-deformation curves during VV tests. The force–deformation curves from the first trial of the constrained VV tests (CT, *thin grey dotted line*) and unconstrained VV tests using FPH (*thin black dash*-*dotted line*), FPA (*thick grey dashed line*) and FPHFM (*thick line*) at (**a**) 0° and (**b**) 30° flexion. FPA and FPHFM had similar measured stiffness both for valgus/varus both at 0° and 30° knee flexion during AP tests while those measured using CT and FPH were different. During CT where significant constraining forces and moments were required, the target moment was reached with smaller angular displacement, indicating less laxity, when compared to those of the unconstrained tests using the three unconstrained control methods

Anisotropy was found in the AP stiffness of the knee joint as indicated by the non-symmetric force–deformation curves about the neutral position while the phenomenon was less obvious for VV stiffness (Fig. 15). For the force–deformation curves during the AP test at 0° knee flexion, the maximum anterior deformation was close to 15 mm and the maximum posterior deformation was −7.5 mm (Fig. 14a). At 30° flexion, the values were decreased to around 12 mm for anterior deformations (Fig. 14b). For both 0° and 30° flexion, the force–displacement curves between CT and unconstrained anterior drawer tests were significantly different, and those measured by FPH were also different from those by FPA and FPHFM methods (Fig. 14). These differences were less apparent during posterior drawer tests (Fig. 14). For the force–displacement curves during the VV test at 0° knee flexion, the maximum varus angle was close to 6 degrees and the maximum valgus angle was 5 degrees (Fig. 15a). At 30° flexion, the values were decreased to around 5° for varus angle and were increased to around 7° for valgus angle (Fig. 15b). For both 0° and 30° flexion, the force–displacement curves between CT and unconstrained tests were significantly different, and those measured by FPH were also different from those by FPA and FPHFM methods (Fig. 15).

## Discussion

The purposes of the current study were to develop a robot-based biological joint-testing system (RJTS) and to evaluate two new hybrid control methods (FPA, FPHFM) and an existing method (FPH) for their performance on unconstrained AP and VV laxity tests of a human knee joint. All the control methods were shown to be repeatable as indicated by the small standard deviations of the repeated measurements. The FPHFM was found to have the best performance in reducing the constraining forces and moments for all tests, while FPA was slightly better than FPH for AP tests, but worse for VV tests. However, FPHFM and FPA achieved better accuracy at the expense of additional control iterations, and thus increased total time. It is suggested that the current results will be useful for selecting a force-position hybrid control method for unconstrained laxity testing of biological joints using an industrial robot-based testing system.

During the constrained AP test using the RJTS, large constraining forces and moments were needed in the secondary DOFs in order to maintain accurate translation along the primary AP axis (Figs. 10, 11). These results were in agreement with those reported in the literature [17, 29]. On the other hand, the current constrained VV tests on the knee joint using the RJTS were the first of their kind to be reported. Large PD forces were needed to facilitate the rotation about the AP axis when subject to increasing VV moments (Figs. 12, 13). In contrast to constrained control, the unconstrained control methods ensured that the constraining forces and moments for the secondary DOFs were close to zero, allowing unresisted movement of the tibia when subject to the primary test loads (AP force or VV moments). During the unconstrained AP test using FPH, the force and moment curves of the primary and secondary DOFs (Figs. 10, 11) were in good agreement with those reported in previous studies [17, 29]. These results indicate that the current RJTS has the same function as the system reported by Fujie et al. [17, 29], and thus could serve as a platform for comparing the performance of different control methods for unconstrained laxity tests.

The FPHFM control method showed the best performance among the tested control methods in the unconstrained AP and VV tests, as indicated by the smallest RMS values of the residual constraining PD and LM forces, and VV and IE moments. The RMS values of the residual PD and LM force, and the VV and IE moment components were used to evaluate the methods because the residual forces and moments in the secondary DOF other than along the primary axis and FE angle had to be kept zero during the unconstrained laxity tests. Based on the RMS values of the residual force and moment components, the FPA appeared to be better than the FPH for AP tests, but worse for VV tests. Further examination of the characteristics of the control methods will help reveal the reasons for the observed outcome.

The performance of the FPH appeared to be related directly to the choice of the C-matrix. The residual constraining forces and moments for the unconstrained DOF during the current step were used to calculate the infinitesimal displacements corresponding to these secondary DOFs for the next step using the selected C-matrix. This approach attempted to reduce the constraining forces and moments for the next step but did not correct the non-zero constraining forces/moments during the current step. Moreover, if the knee stiffness were to be changed at the new step, the predicted positions and orientations based on the residual constraining forces and moments from the previous step would not completely remove the residual forces and moments during the current step. This approach helped ensure the convergence of the algorithm but was not effective in reducing the constraining forces and moments in the secondary DOFs. In the current study, the same C-matrix was selected for both 0**°** and 30**°** knee flexion. As has been reported in the literature, the stiffness of the knee joint varies over the knee flexion range [32]. Therefore, the compliance of the joint is fundamentally different at different flexion angles. The differences in the RMS of the residual force and moment components between 0**°** and 30**°** knee flexion suggest that the selected C-matrix may have contributed to the observed differences in the accuracy of the tests using the FPH.

The additional force-moment control iterations at each increment of the displacement in the primary DOF of the FPHFM were found to be effective in reducing the constraining forces and moments simultaneously for the current position, giving better accuracy and precision. With the alternate position/force control strategy in FPA, the force-moment control following the previous position control was applied for reducing the constraining forces and moments, but caused another positional bias from the target values. On the other hand, the FPHFM and FPA were less sensitive to the choice of the C-matrix because any error associated with the C-matrix would be corrected by the recursive feedback for each increment. As expected, the differences between FPHFM and FPA appeared to be related mainly to the control strategies adopted by the methods in the feedback loop for each increment.

The total time of the hybrid control methods was related to the number of control iterations used to converge to the zero residual point. Since both FPA and FPHFM needed additional force-moment control iterations to reduce the non-zero constraining forces and moments for each AP position increment, given the same increment of the primary displacement, the total time for FPH for a single laxity test was significantly smaller than those of the other two methods. Given the same compliance matrix, FPHFM and FPA reduced the residual constraining loads of FPH at the expense of additional control iterations, and thus increased total time, the FPA being about 10 % longer than FPHFM. However, the increased number of control iterations and the total time in the FPHFM may not necessarily be a drawback of the method. This is because the ligaments and the surrounding soft tissues are visco-elastic materials. Therefore, the mechanical response of the ligaments depends on the speed of the applied force or displacements. With the FPHFM, since several additional iterations—and thus time—would be taken to reduce the constraining force or moment components, the time dependency of the force–deformation characteristics could be reduced with the increased time for each position increment. The effects of viscosity can also be reduced by adding a pause between steps for FPH or any other control methods, although the steps may not be the converged steps (i.e., zero constraining forces and moments). Nonetheless, since the current control methods are all stepwise control methods for simulating clinical laxity tests using industrial robots, and the tests are performed at a relatively low loading rate [4, 21, 26], the size of residual constraining forces and moments are considered more important than the differences in the total time used.

The measured AP and VV force–displacement curves were significantly affected by the residual constraining forces and moments, as indicated the RMS values (Tables 2, 3) and the maximum tibial displacements corresponding to the target force/moment (Figs. 14, 15). During CT where significant constraining forces and moments were required, the target force/moment was reached with smaller displacement, indicating less laxity, when compared to those of the unconstrained tests using the three unconstrained control methods (Figs. 14, 15). Among the unconstrained control methods, a similar trend was also found: the smaller the RMS values of the constraining forces and moments, the bigger the maximum displacement, and thus the joint laxity. These results suggest that the control of the RJTS to reduce as much as possible the constraining forces and moments during unconstrained laxity tests for more accurately determining the joint stiffness characteristics is essential for various clinical applications, such as establishing baseline data for normal joint biomechanics [15–17, 24, 29, 30], exploring injury biomechanics (e.g., ligament ruptures, [18]), and evaluating existing and new treatment methods (e.g., reconstructed ligaments and total knee replacements [7, 18]. From the current results, it appears that FPHFM and FPA are capable of improving FPH in reducing the constraining forces and moments, but the FPHFM is better than FPA when considering both the residual constraining loads and the total control time.

The current study was limited to evaluating three hybrid control methods. The findings may be applied to similar control methods, but for other types of control methods further study will be needed. Another limitation was that only the AP and VV tests at 0**°** and 30**°** knee flexion were considered. These tests were chosen because they corresponded to the common laxity tests in clinical settings. With proper settings of the control parameters, the current RJTS can easily be configured to perform laxity tests in other directions and knee flexion positions in future studies. Another factor that affected the accuracy of the control methods was the values of the infinitesimal displacement. The residual constraining force or moment components could also be reduced with the reduced infinitesimal displacements for the three control methods.

## Conclusions

As opposed to traditional constrained tests, all three control methods successfully reduced the constraining forces and moments for both unconstrained AP and VV tests, FPHFM being the best followed in order by FPA and FPH. Given the same compliance matrix, FPHFM and FPA reduced the residual constraining loads of FPH at the expense of additional control iterations, and thus increased total time, the FPA being about 10 % longer than FPHFM. The current findings suggest that the FPHFM would be the best choice among the methods considered when accurate unconstrained laxity testing is critical and longer total time is acceptable in the intended clinical applications. The current results will be useful for selecting a force-position hybrid control method for unconstrained laxity testing of biological joints using an industrial robot-based testing system.
